# Zinc ions attenuates iridovirus infection through regulation of ferroptosis pathways

**DOI:** 10.1038/s41420-026-03114-x

**Published:** 2026-04-20

**Authors:** Yanlin You, Mincong Liang, Xueqian Cao, Weiqiang Pan, Jian He, Kun Wu, Shaoping Weng, Jianguo He, Changjun Guo

**Affiliations:** 1 School of Life Sciences, China-ASEAN Belt and Road Joint Laboratory on Mariculture Technology & State Key Laboratory for Biocontrol, Guangdong Province Key Laboratory for Aquatic Economic Animals, Sun Yat-sen University, Guangzhou, China; 2 School of Marine Sciences, Southern Marine Science and Engineering Guangdong Laboratory (Zhuhai), Guangdong Provincial Observation and Research Station for Marine Ranching of the Lingdingyang Bay, Sun Yat-sen University, Guangzhou, China

**Keywords:** Viral infection, Cell death

## Abstract

Zinc is widely acknowledged as an inducer of ferroptosis, a type of regulated cell death process. Generally, ferroptosis is known to facilitate viral replication. However, an intriguing aspect is that despite its role in inducing ferroptosis, zinc demonstrates broad-spectrum antiviral activity. This study focused on investigating how zinc ions inhibit the replication of the infectious spleen and kidney necrosis virus (ISKNV; an iridovirus) in mandarin fish (a teleost fish) through zinc influx-dependent ferroptosis activation. RNA-seq, enzyme activity assays, and transmission electron microscopy results revealed that zinc could promote ferroptosis in fish. Single-cell sequencing and in vivo validation confirmed that ISKNV infection triggered host ferroptosis, while pharmacological modulation showed that ferroptosis suppressed viral infection. Delving deeper into the mechanism, it was discovered that the expression of *GPX4* and *xCT* partially reversed the antiviral effect of zinc, suggesting that the xCT/GPX4 axis mediated ferroptosis is a mechanistic participant. In vivo challenge assays further verified that zinc monotherapy reduced mortality by 25% and delayed disease progression, and these effects were abolished by co-treatment with the ferroptosis inhibitor vitamin C. This work sheds light on a zinc-ferroptosis axis as an unconventional antiviral defense, redefining both zinc’s immune mechanism and the dual role of ferroptosis in viral infection.

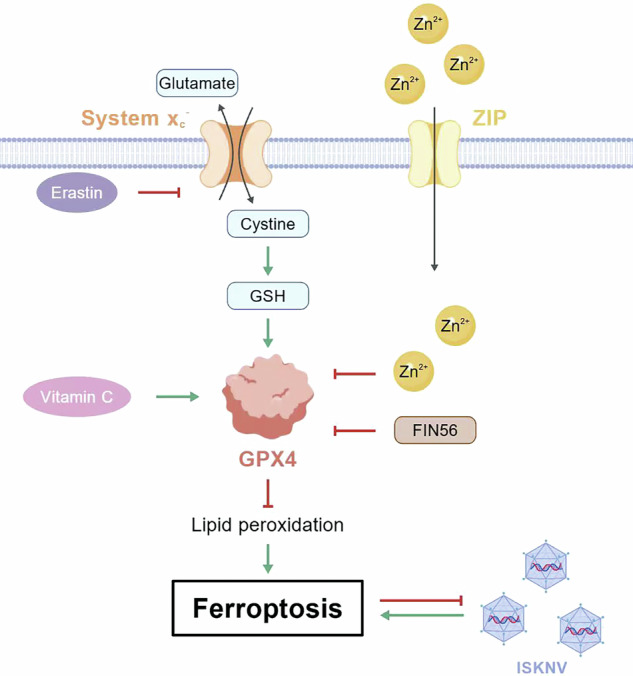

## Introduction

Disease pathogenesis is a result of dynamic interactions between pathogens and the host’s immune system, which are modulated by multifactorial regulators, including essential trace elements [1, 2]. Zinc, a crucial micronutrient, plays a pivotal role in orchestrating diverse immune processes, such as anti-inflammatory [3], anti-aging [4], antitumor [5], and antiviral [6] activities. In vitro studies have shown that zinc can inhibit viral polyprotein processing, thereby exhibiting antiviral activity against picornavirus [7], foot-and-mouth disease virus [8], and rhinovirus [9]. Clinical trials have further demonstrated that zinc supplementation is associated with attenuated symptoms and a shortened duration of common colds [10–12]. Although these clinical findings support the antiviral potential of zinc, the underlying mechanisms remain largely unknown.

Cells maintain zinc levels within a narrow, safe range via zinc homeostasis, primarily controlled by zinc transporters (ZIP and ZnT proteins), that regulate zinc inflow and outflow [13]. Metal regulatory transcription factor 1 (MTF1) is a key regulator and primary zinc sensor. When zinc levels rise, MTF1 enters the nucleus, binds to Metal response elements (MREs), and activates genes involved in zinc detoxification and redistribution, like metallothioneins [14]. Though essential, excessive zinc intake is cytotoxic, reducing cell viability and causing cell death. Zinc is implicated in inducing ferroptosis in various cell types [15–17]. Ferroptosis is a regulated cell death form, iron-dependent, and with distinct biochemical mechanisms from apoptosis and autophagy [18]. Ferroptosis is strongly linked to viral infections. Most viruses exploit ferroptosis to boost replication and dissemination, aiding transmission and harming host organs [19], such as HIV and SARS-CoV-2. This presents a contradictory phenomenon: How does zinc, a ferroptosis activator, have antiviral effects instead of accelerating viral spread?

Iridoviruses, a family of large double-stranded DNA viruses, seriously threaten global aquaculture and aquatic ecosystems [20]. Infectious spleen and kidney necrosis virus (ISKNV), a member of iridovirus, is especially worrisome. It has an exceptionally broad host range, being found in various fish species from at least 7 orders and 22 families [21, 22]. ISKNV outbreaks spread rapidly and cause high mortality, leading to huge economic losses. Surveillance has found ISKNV-like viruses in ornamental fish in over ten countries [23], showing their widespread and hidden spread. Besides harming aquaculture, ISKNV also risks infecting wild species in these countries [21, 22], threatening aquatic biodiversity. Its global distribution, fueled by trade, makes it a persistent and widespread pathogen.

In this study, we revealed that zinc exerted potent antiviral effects against ISKNV infection by inducing ferroptosis in a manner dependent on zinc influx. These findings will contribute to a better understanding of the dual role played by the zinc-ferroptosis axis in antiviral immunity and pro-viral replication.

## Results

### Zinc ions effectively inhibits ISKNV replication

**Fig. 1:**
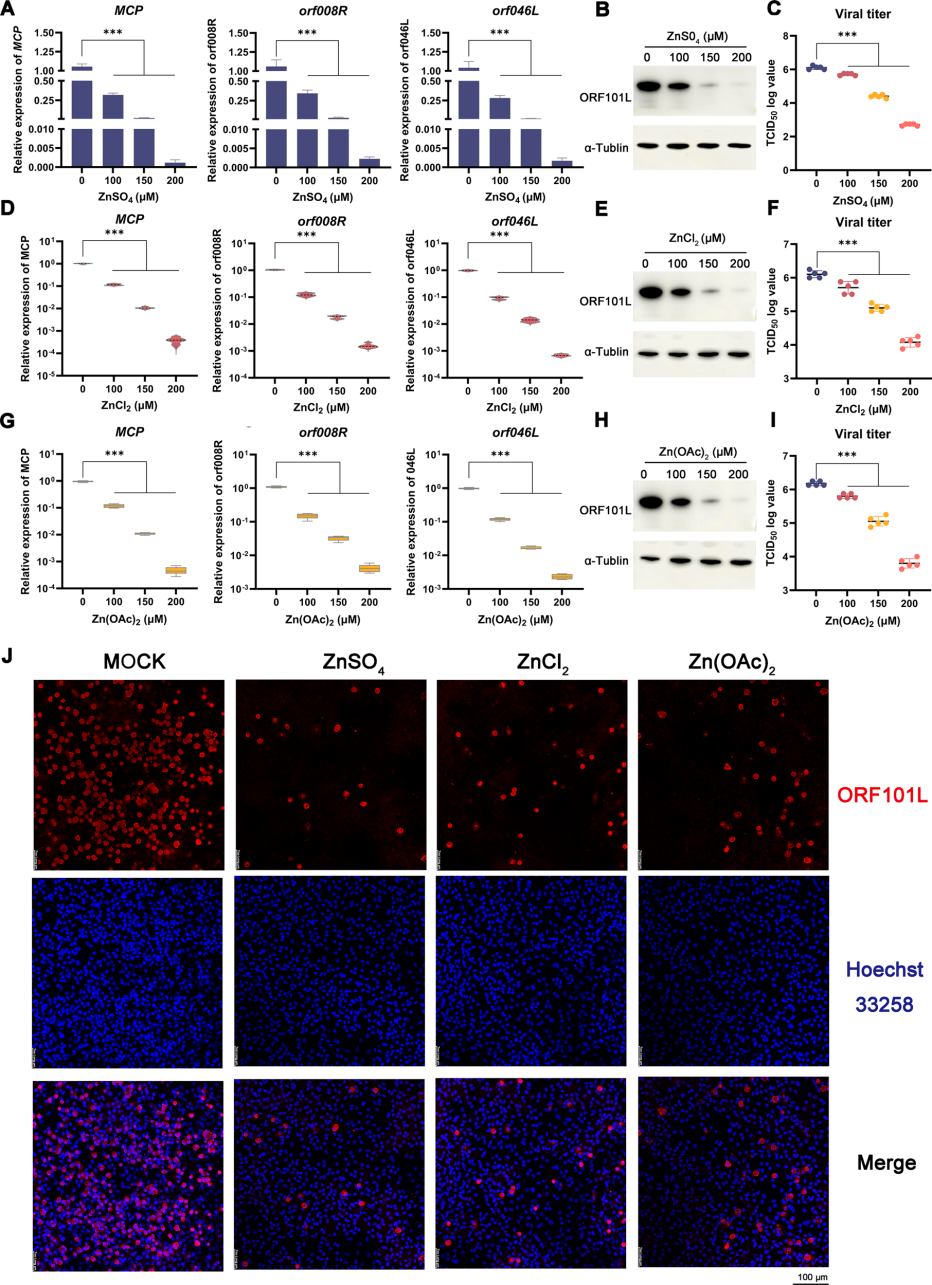
Zinc induces an antiviral state. **A**–**J** Zinc treatment inhibits ISKNV replication. MFF-1 cells were infected with ISKNV for 4 h, then treated with indicated concentrations of ZnSO_4_, ZnCl_2_, or Zn(OAc)_2_. Viral gene expression was assessed by RT–qPCR (**A**, **D**, **G**) and Western blot (**B**, **E**, **H**). Viral titers were determined by the TCID_50_ assay (**C**, **F**, **I**) at 72 hpi. **J** All three zinc salts reduce viral protein synthesis. Immunofluorescence analysis showing ORF101L (red) in infected cells treated with 200 μM of each zinc compound. Nuclei were counterstained with Hoechst 33258 (blue). Scale bar: 100 μm. Data are shown as mean ± SD (*n* = 5). ***P < 0.001.

To investigate the role of zinc ions in ISKNV infection, MFF-1 cells were infected and then treated with ZnSO_4_ at concentrations determined to be non-cytotoxic by a CCK-8 assay (Fig. S1). Treatment was maintained for 72 h. Analysis of viral infection by RT–qPCR (viral gene expression), Western blot (viral protein levels), and TCID_50_ assays (viral titers) consistently revealed a significant, dose-dependent inhibition of ISKNV replication by ZnSO_4_ (Fig. 1A–C). These findings indicated that zinc sulfate effectively inhibits ISKNV replication.

To confirm that the observed inhibition was mediated specifically by Zn^2+^ rather than the counter-anion, we tested two additional zinc salts with different anions: zinc chloride (ZnCl_2_) and zinc acetate (Zn(OAc)_2_). Both compounds similarly suppressed ISKNV replication across all assays in a dose-dependent manner (Fig. 1D–I). Furthermore, indirect immunofluorescence assay (IFA) confirmed that all three salts markedly reduced both the number of infected cells and the intensity of viral protein staining compared to the infected control (Fig. 1J). Given that Zn^2+^ is the common component among the three salts, these results establish that the antiviral activity is specifically attributable to zinc ions, not the associated anions. Collectively, these data demonstrate that zinc ions exert potent antiviral activity against ISKNV, significantly reducing viral load and infectious progeny in a dose-dependent manner.

### Zinc-mediated antiviral response depends on the influx of zinc ions

**Fig. 2:**
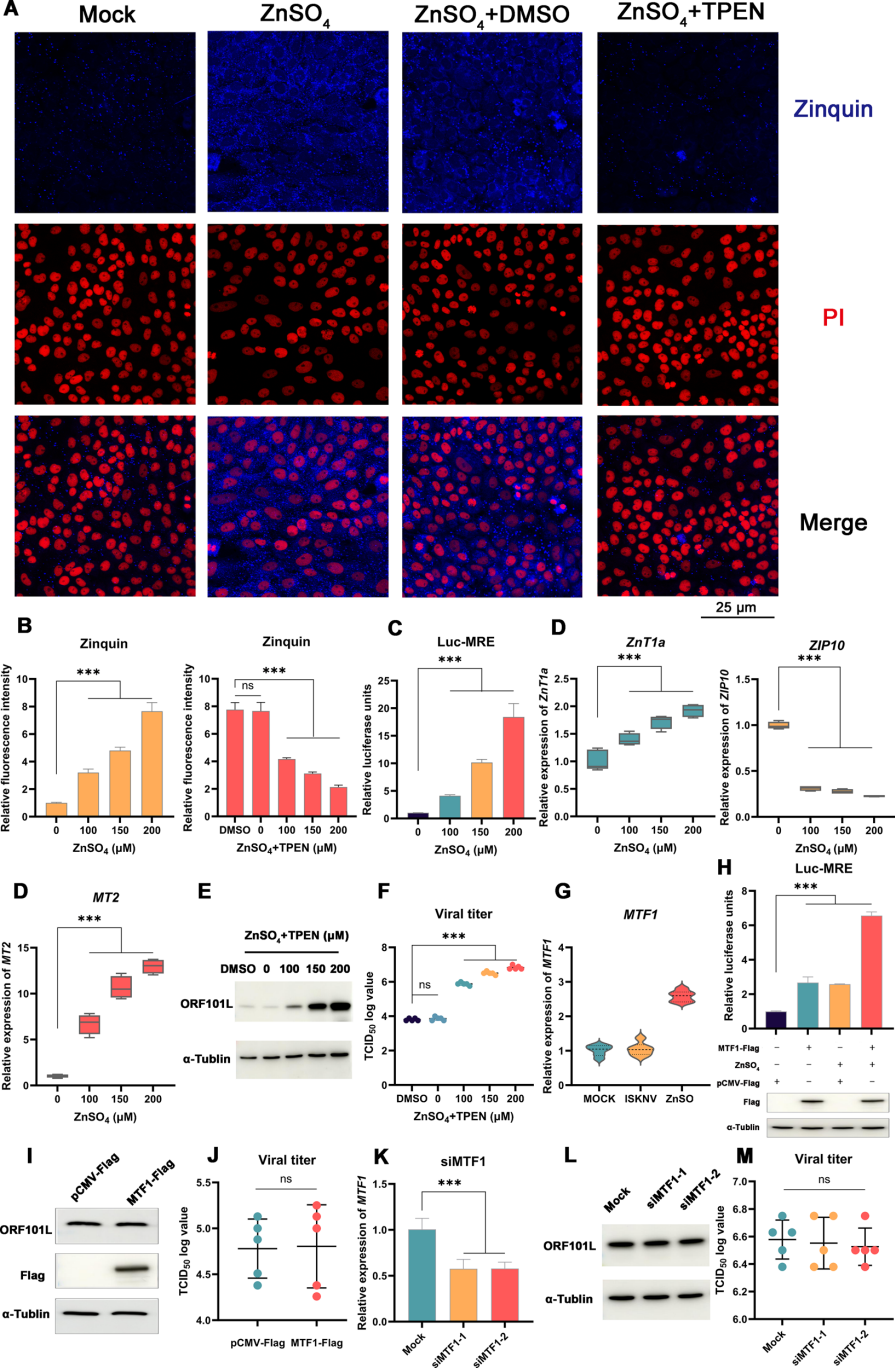
The zinc-mediated antiviral response depends on the influx of zinc ions. **A**, **B** Zinc elevates intracellular free zinc levels. Zinquin ethyl ester staining (blue) shows free zinc in cells treated with ZnSO_4_ ± TPEN. Nuclei are stained with PI (red). Scale bar: 25 μm. **B** Quantification of fluorescence intensity. **C** Zinc activates MRE-dependent transcription. Luciferase reporter assay in cells transfected with Luc-MRE and treated with ZnSO_4_. **D** Zinc upregulates zinc homeostasis genes. Expression of *ZnT1a*, *ZIP10*, and *MT2* after ZnSO_4_ treatment. Zinc chelation abrogates zinc-mediated antiviral activity. Viral protein levels (**E**) and viral titers (**F**) in infected cells co-treated with ZnSO_4_ and TPEN. DMSO served as the solvent control. **G** *MTF1* expression is not altered by ISKNV infection. *MTF1* expression levels in ISKNV-infected and ZnSO_4_-treated cells at 72 hpi. **H** *MTF1* overexpression activates MRE signaling. Luc-MRE activity in cells transfected with *MTF1*-Flag and/or treated with ZnSO_4_. *MTF1* overexpression does not affect ISKNV replication. Viral protein levels (**I**) and viral titers (**J**) in infected cells transfected with *MTF1*-Flag or an empty vector control. *MTF1* knockdown does not affect ISKNV replication. **K** Validation of efficient *MTF1* knockdown. Viral protein levels (**L**) and viral titers (**M**) in infected cells transfected with *MTF1*-targeting or non-targeting control siRNA (Mock). Data are shown as mean ± SD (*n* = 5). ***P < 0.001; *ns*, not significant.

To elucidate the mechanism underlying zinc-mediated antiviral responses, we initially assessed the influx of zinc ions. In MFF-1 cells, treatment with ZnSO_4_ induced a dose-dependent increase in intracellular free Zn^2+^, visualized using the fluorescent probe Zinquin (Fig. 2A, B). This signal was abrogated by co-treatment with the specific zinc chelator TPEN. We further confirmed functional zinc influx using a dual-luciferase reporter assay, which showed that ZnSO_4_ robustly activated transcription from MREs (Fig. 2C). Consistent with MRE activation, RT–qPCR analysis confirmed that the expression of zinc-responsive genes (*ZnT1a*, *ZIP10*, and *MT2*) was significantly altered by zinc treatment (Fig. 2D). These data demonstrate that exogenous zinc triggers a canonical intracellular zinc-signaling response. Crucially, this zinc influx was essential for the antiviral activity, as co-treatment with TPEN dose-dependently reversed the inhibition of ISKNV replication by ZnSO_4_ in both Western blot and TCID_50_ assays (Fig. 2E, F).

Given the observed zinc influx and MRE activation, we next investigated the role of the major zinc-sensing transcription factor MTF1. While zinc treatment increased *MTF1* expression, ISKNV infection itself did not alter *MTF1* levels (Fig. 2G). Although overexpression of *MTF1*-Flag activated the MRE reporter (Fig. 2H), modulating MTF1 activity had no effect on viral replication: neither *MTF1*-Flag overexpression (Fig. 2I, J) nor siRNA-mediated *MTF1* knockdown (Fig. 2K–M) affected viral protein levels or progeny titers. Collectively, these results indicate that while zinc influx is essential for the antiviral effect, the downstream MTF1 signaling axis is dispensable for the inhibition of ISKNV.

### Zinc triggers ferroptosis

**Fig. 3:**
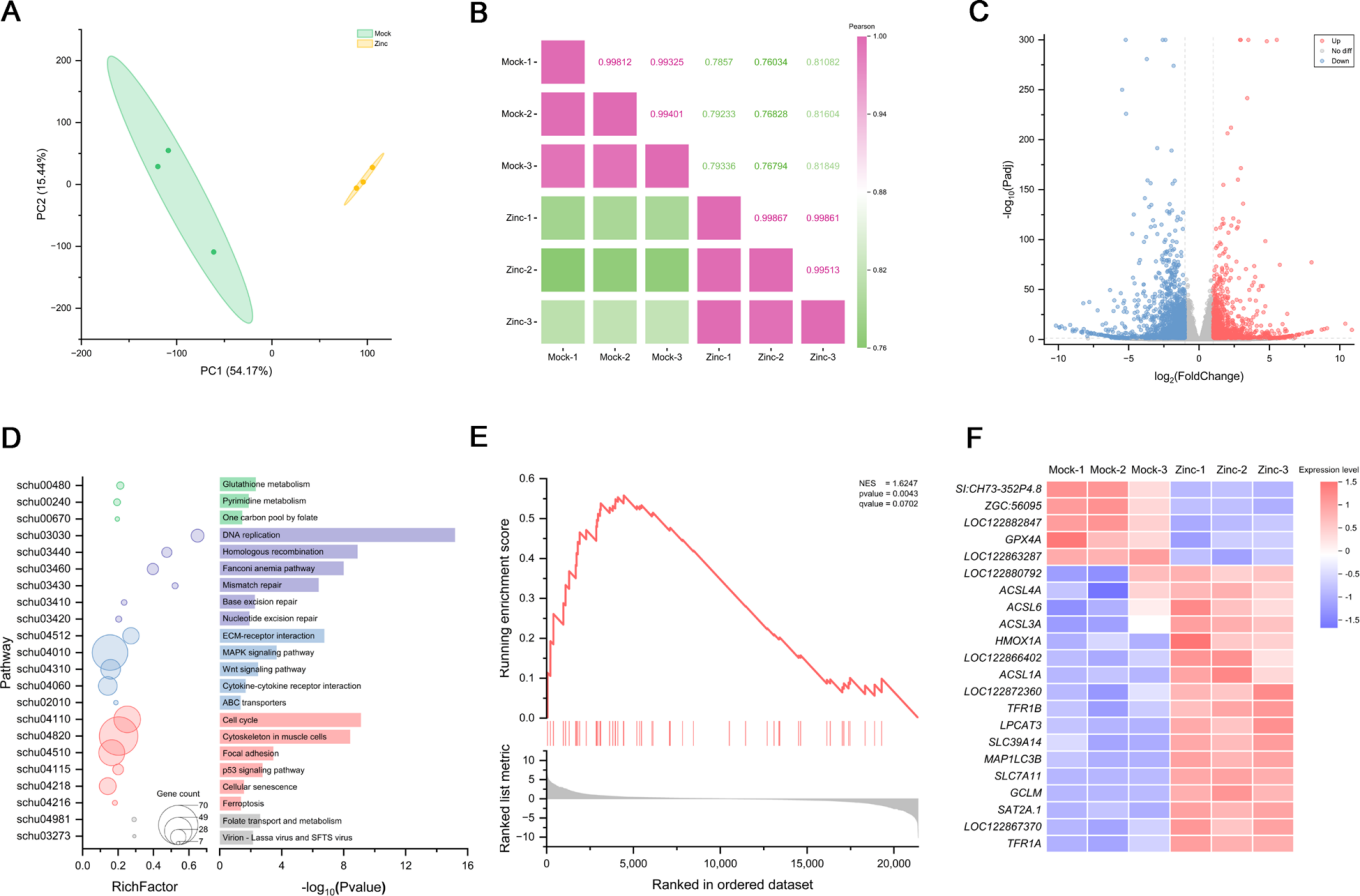
Transcriptome analysis after treatment with zinc sulfate. **A** Principal component analysis results of the samples. **B** Intra-group correlation analysis results of the samples. **C** Volcano plot showing DEGs between Mock and Zinc, with red representing upregulated DEGs and blue representing downregulated DEGs. **D** KEGG pathway enrichment of DEGs between Mock and Zinc. **E** GSEA analysis results of the ferroptosis pathway. **F** Expression heatmap of DEGs in the ferroptosis pathway between Mock and Zinc.

To seek alternative mechanisms underlying zinc-mediated ISKNV suppression, transcriptome sequencing (RNA-seq) analysis of ZnSO4-treated cells was employed. Following 72 h of ZnSO_4_ stimulation in MFF-1 cells, alterations in gene expression were observed between the control (Mock) and stimulated groups (Zinc; Fig. 3A, B). A total of 2 362 differentially expressed genes (DEGs) were identified, comprising 991 upregulated and 1371 downregulated genes (Fig. 3C). KEGG enrichment highlighted significant pathways including MAPK, p53, and Wnt signaling, alongside a marked activation of the ferroptosis pathway (Fig. 3D). Further investigation using gene set enrichment analysis (GSEA) confirmed a significant upregulation trend in the ferroptosis-related gene set (Fig. 3E). A heatmap visualization further illustrated the relative expression levels of key ferroptosis-associated genes across samples (Fig. 3F).

**Fig. 4:**
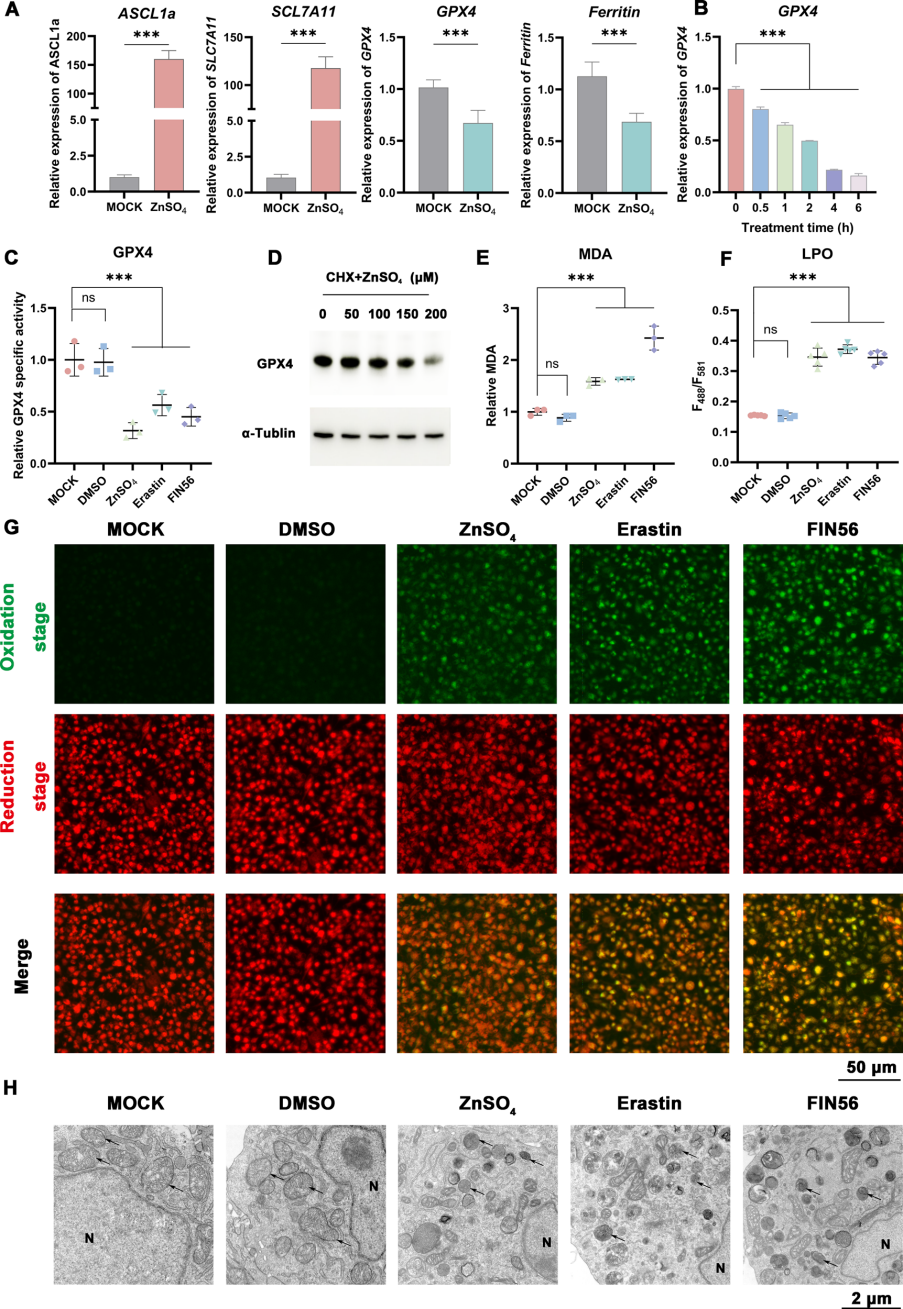
Zinc triggers ferroptosis. **A**, **B** Zinc regulates ferroptosis-related gene expression. mRNA levels of *SLC7A11*, *ACSL1a*, *Ferritin*, and *GPX4* after ZnSO_4_ treatment. Zinc suppresses GPX4 function. **C** GPX4 activity is inhibited by ZnSO_4_ treatment. **D** GPX4 protein degradation is accelerated by co-treatment with ZnSO_4_ and cycloheximide (CHX). Zinc enhances malondialdehyde (MDA) (**E**) and lipid hydroperoxides (LPO) levels (**F**, **G**). C11-BODIPY^581/591^ staining (**G**) showing LPO (green: oxidized; red: reduced). Scale bar: 100 μm. Quantification in (**F**). Erastin and FIN56 as positive controls. DMSO as solvent control. **H** Zinc triggers mitochondrial damage. TEM images of mitochondria (black arrows) after ZnSO_4_ treatment. Erastin and FIN56 as positive controls. DMSO as solvent control. N, nucleus. Scale bar: 2 μm. Data are shown as mean ± SD (*n* ≥ 3). ***P < 0.001; *ns*, not significant.

Consistent with the sequencing data, ZnSO_4_ upregulated the expression of *SLC7A11* (*xCT*) and *ACSL1a*, while downregulating those of *Ferritin* and *GPX4* (Fig. 4A, B). Correspondingly, ZnSO_4_ treatment induced a dose-dependent decrease in GPX4 enzyme activity (Fig. 4C). Further supporting the loss of GPX4 function, co-treatment with the translation inhibitor cycloheximide (CHX) and ZnSO_4_ reduced GPX4 protein levels (Fig. 4D), indicating that zinc also promotes GPX4 protein degradation. These changes were accompanied by the marked accumulation of the lipid peroxidation products malondialdehyde (MDA) and lipid hydroperoxides (LPO) (Fig. 4E–G). The increase in LPO was visually confirmed using the fluorescent probe C11-BODIPY^581/591^, which showed a shift in fluorescence comparable to that induced by the canonical ferroptosis inducers Erastin and FIN56 (Fig. 4F, G). Finally, transmission electron microscopy revealed the characteristic ultrastructural hallmarks of ferroptosis in ZnSO_4_-treated cells, including mitochondrial shrinkage, increased membrane density, and loss of cristae (Fig. 4H). Collectively, these integrated multi-omics, biochemical, imaging, and ultrastructural data establish that zinc triggers ferroptosis.

### ISKNV infection reconfigures the cellular landscape of the host spleen

**Fig. 5:**
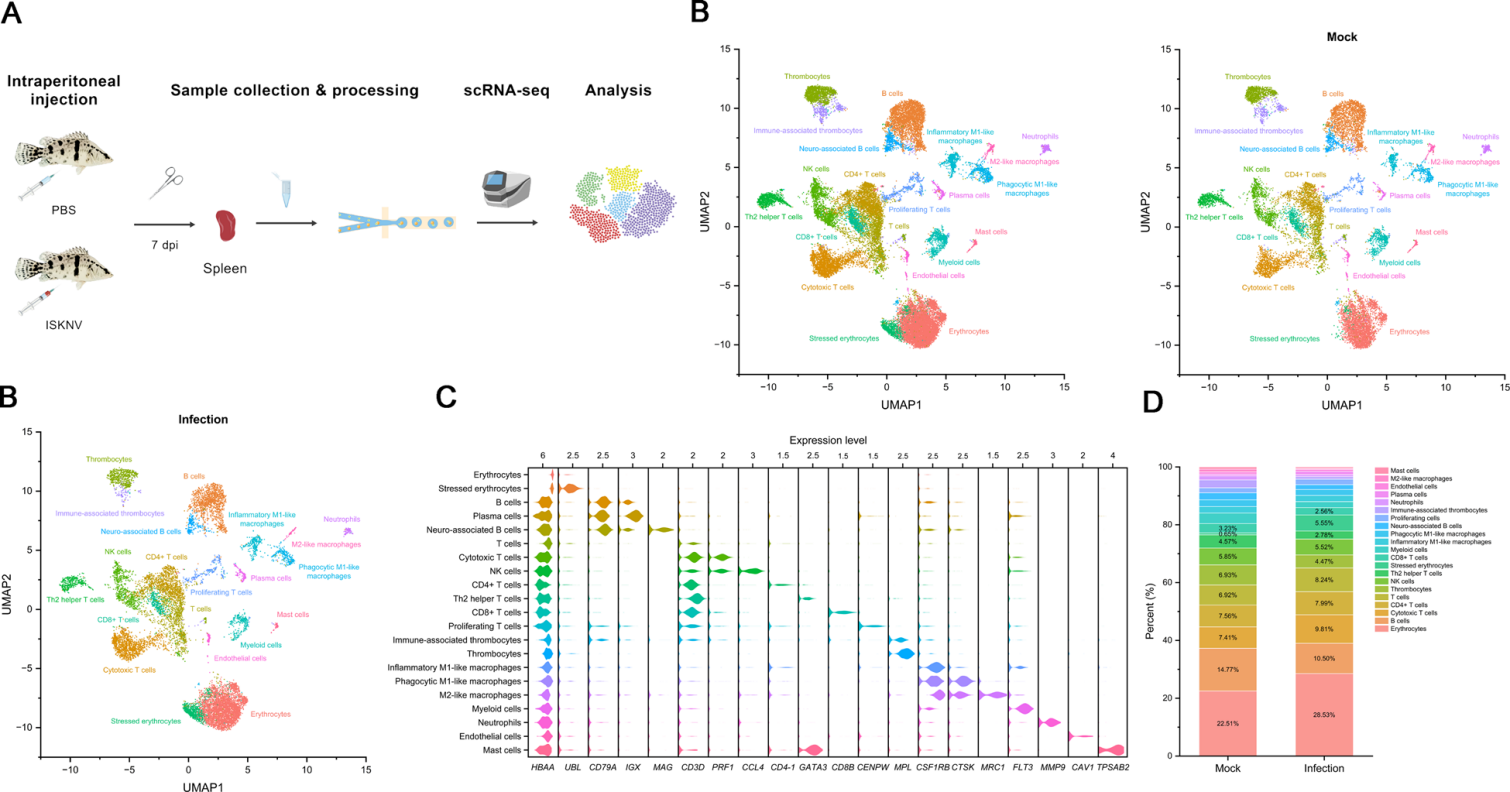
ISKNV mediates changes in the composition of host spleen cell populations. **A** Sample preparation and scRNA-seq analysis workflow. **B** UMAP plot of 21 cell clusters in the spleen of control and infected mandarin fish, each dot represents a cell, and each color represents a cell cluster. **C** Expression of selected marker genes in 21 cell clusters in the mandarin fish spleen. **D** Proportions of 21 cell clusters in the spleen of control and infected mandarin fish.

To investigate whether ISKNV infection itself, independent of exogenous zinc exposure, induces ferroptosis in host tissues, we performed single-cell RNA sequencing (scRNA-seq) on spleens isolated from infected fish at 7 dpi (Fig. 5A). The spleen was selected as a major target organ for ISKNV replication [21]. A total of 14 688 cells from the control group and 13 650 cells from the infected group were analyzed, resulting in 21 transcriptionally distinct cell clusters (Fig. 5B). These included erythrocytes, stressed erythrocytes, B cells, plasma cells, T cells, cytotoxic T cells, NK cells, CD4^+^ T cells, Th2 helper T cells, CD8^+^ T cells, proliferating T cells, immune-associated thrombocytes, thrombocytes, inflammatory M1-like macrophages, phagocytic M1-like macrophages, M2-like macrophages, myeloid cells, neutrophils, endothelial cells, and mast cells. Key marker genes defining these splenic cell populations are depicted in Fig. 5C.

Notably, we identified a distinct subpopulation in erythrocytes, named “stressed erythrocytes”, which expanded markedly upon ISKNV infection—increasing from 0.65% in controls to 5.55% in infected spleens (Fig. 5D). UMAP visualization confirmed that this cluster was predominantly located within the infected group (Fig. 5B), suggesting a potential role in the host response to viral infection. In addition to exhibiting high expression of erythroid-specific genes such as hemoglobin, these stressed erythrocytes specifically and strongly expressed a polyubiquitin-like gene (Gene ID: 122876799). This observation implies a possible link to ISKNV’s strategy of exploiting ubiquitination to evade host immunity [24] and further suggests that erythrocytes in mandarin fish spleen may participate in the antiviral immune response.

### Ferroptosis responds to viral infection in vivo

**Fig. 6:**
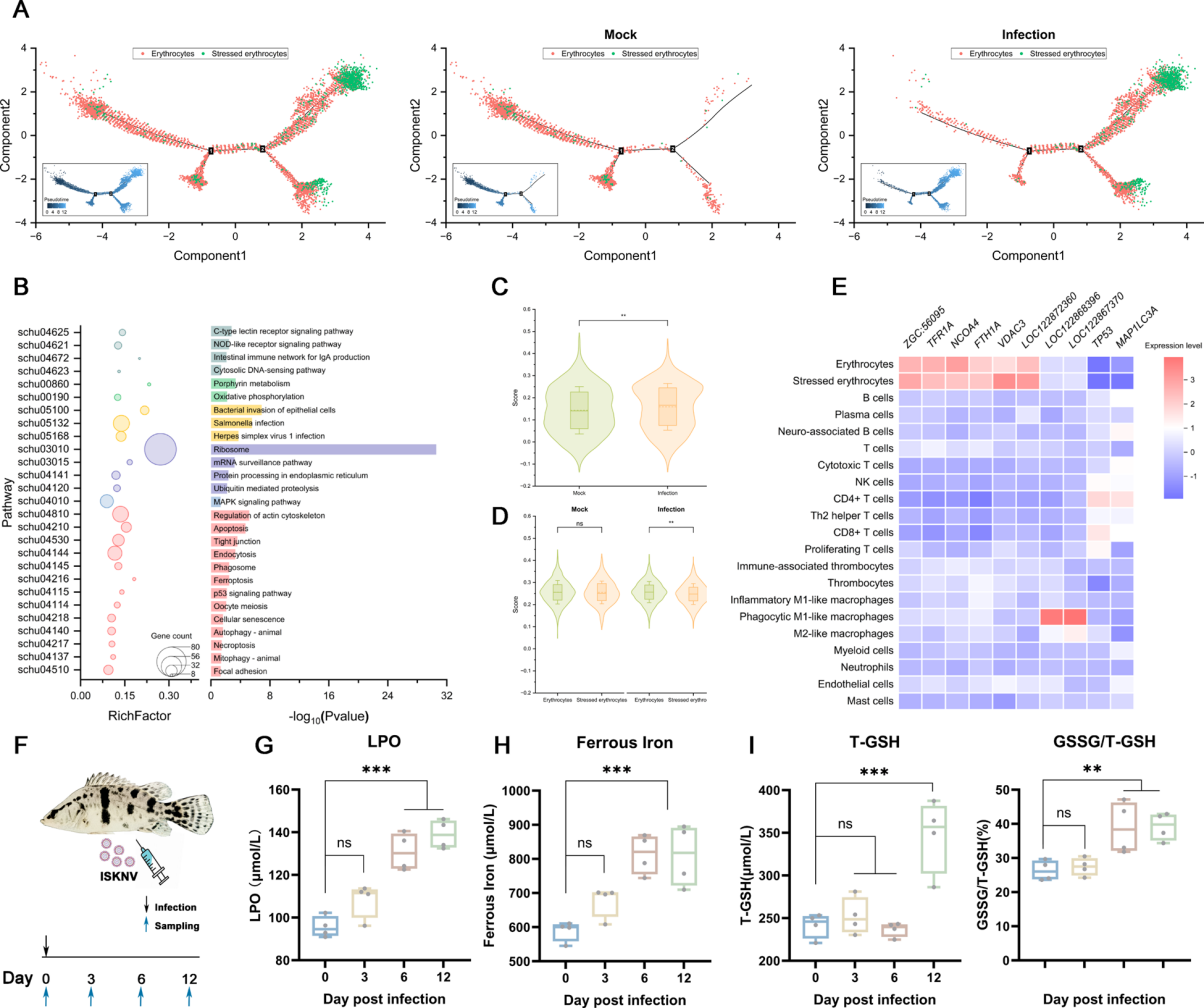
Ferroptosis responds to viral infection in vivo. Pseudotime analysis and DEGs enrichment analysis of erythrocytes in the spleen of mandarin fish after ISKNV infection. **A** Pseudotime analysis of erythrocytes in the spleen of control and infected mandarin fish, each dot represents a cell, and each color represents a cell cluster. **B** KEGG pathway enrichment of DEGs in stressed erythrocytes in the mandarin fish spleen. **C** Overall ferroptosis biological process scores in spleen cells of control and infected mandarin fish. **D** Ferroptosis biological process scores in normal erythrocytes and stressed erythrocytes in the spleen of control and infected mandarin fish. **E** Expression heatmap of DEGs in the ferroptosis pathway across different cell clusters in the mandarin fish spleen. **F** Schematic diagram of the experimental timeline and procedures. **G–I** Time-course changes in ferroptosis markers (LPO, Fe^2+^, T-GSH, GSSG) in blood after ISKNV infection. Data are shown as mean ± SD (*n* ≥ 3). **P < 0.01; ***P < 0.001; *ns*, not significant.

To further investigate whether the heterogeneity of erythrocytes in the mandarin fish spleen is associated with viral infection, we examined the infection-induced erythroid differentiation dynamics by pseudotime analysis using Monocle2. The trajectory analysis revealed that stressed erythrocytes originated from erythrocytes, displaying a clear differentiation path from uninfected to infected states. Cells from the Mock group were predominantly positioned at the early stage of the trajectory, whereas those from the infected group showed a pronounced shift toward the stressed erythrocyte state (Fig. 6A). This distinct divergence was not observed in the pseudotime trajectories of other immune cell types, including B cells, T cells, thrombocytes, and macrophages (Fig. S2), indicating that the observed erythroid heterogeneity is specifically driven by ISKNV infection.

KEGG enrichment analysis of DEGs in stressed erythrocytes highlighted several significantly enriched immune-related pathways, including apoptosis, the C-type lectin receptor signaling pathway, phagosome, the p53 signaling pathway, the intestinal immune network for IgA production, and the MAPK signaling pathway. Notably, the ferroptosis pathway was also among the significantly enriched terms (Fig. 6B). By leveraging a gene set comprising 55 ferroptosis-related genes defined in the KEGG database, we computed a pathway activity score, which was significantly elevated in cells from the infected group compared to the control (Fig. 6C). At the erythrocyte subpopulation level, while no significant difference in ferroptosis scores was detected between erythrocytes and stressed erythrocytes in control fish, a highly significant increase was observed in stressed erythrocytes from infected fish (Fig. 6D). Consistent with this, multiple key genes associated with the ferroptosis pathway also exhibited altered expression patterns specifically in stressed erythrocytes (Fig. 6E).

To biochemically validate the ferroptosis predicted by scRNA-seq, we longitudinally monitored key markers in host blood during ISKNV infection (Fig. 6F). Levels of lipid hydroperoxides (LPO) and ferrous iron (Fe^2+^) accumulated significantly by 6 days post-infection (dpi) and increased further at 12 dpi (Fig. 6G, H). Concurrently, oxidized glutathione (GSSG) rose markedly at both 6 and 12 dpi, whereas total glutathione (T-GSH) showed a delayed increase only at the later time point (Fig. 6I). This distinct kinetic profile—marked by the early rise in oxidative markers (LPO, Fe^2+^, GSSG) preceding the late increase in total antioxidant capacity (T-GSH)—provides compelling biochemical evidence that ISKNV infection induces systemic ferroptosis in vivo.

### Ferroptosis acts as a defense mechanism to restrict ISKNV replication

**Fig. 7:**
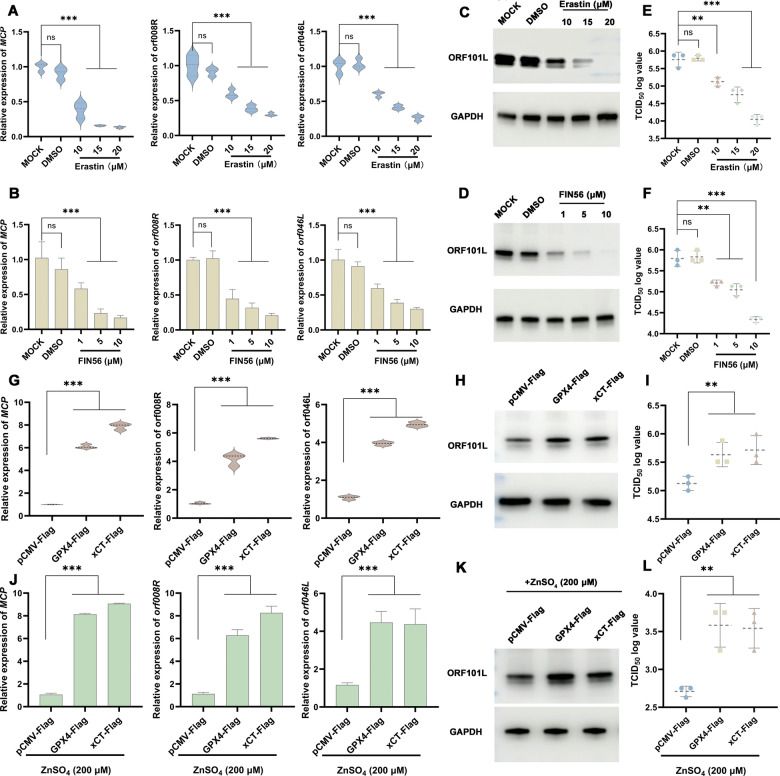
xCT/GPX4-dependent ferroptosis regulates viral replication and is targeted by zinc for antiviral activity. **A**–**F** Ferroptosis inducers inhibit ISKNV replication. Viral gene expression (**A**–**D**) and titers (**E**, **F**) in infected cells treated with Erastin or FIN56. DMSO served as the solvent control. *xCT* or *GPX4* overexpression promotes ISKNV replication. Viral gene expression (**G**, **H**) and titers (**I**) in cells overexpressing *xCT* or *GPX4*. Empty vector pCMV-Flag served as a control. Zinc requires xCT/GPX4 to exert antiviral effects. Viral gene expression (**J**, **K**) and titers (**L**) in ZnSO_4_-treated cells overexpressing *xCT* or *GPX4*. Empty vector pCMV-Flag served as a control. Data are shown as mean ± SD (*n* ≥ 3). **P < 0.01; ***P < 0.001; *ns*, not significant.

Given the concurrent induction of ferroptosis by both zinc and ISKNV infection, we investigated whether ferroptosis intrinsically restricts viral proliferation. To decouple ferroptosis from zinc signaling, ISKNV-infected MFF-1 cells were treated with increasing concentrations of the canonical ferroptosis inducers Erastin or FIN56 for 72 h, with DMSO as the solvent control. Both compounds exerted a potent, dose-dependent inhibition of ISKNV replication, as evidenced by significant reductions in viral gene expression (RT–qPCR), viral protein synthesis (Western blot), and infectious progeny production (TCID_50_) (Fig. 7A–F).

To further establish causality, we genetically modulated core ferroptosis defense pathways. Overexpression of *GPX4*-Flag or *xCT*-Flag (vs. empty vector control) in MFF-1 cells prior to ISKNV infection enhanced viral replication (Fig. 7G–I). This pro-viral effect demonstrates that suppressing ferroptosis machinery (via *GPX4*/*xCT* ectopic expression) creates a permissive environment for ISKNV proliferation. Collectively, these pharmacological and genetic interventions establish ferroptosis as a defense mechanism that directly restricts ISKNV replication.

### Zinc exerts its antiviral activity by inducing ferroptosis via the xCT/GPX4 axis

To definitively establish that zinc-mediated antiviral effects operate via ferroptosis, we performed a genetic rescue experiment targeting the xCT/GPX4 axis—a central ferroptosis regulator [25] whose expression and activity are influenced by zinc (Fig. 4A–D). MFF-1 cells were transfected with *xCT*-Flag, *GPX4*-Flag, or an empty vector control. Following transfection for 24 h, cells were infected with ISKNV and subsequently treated with ZnSO_4_. Viral replication was assessed at 96 hpi. Ectopic expression of either *xCT* or *GPX4* was found to compromise the antiviral activity of ZnSO_4_. Specifically, compared to the empty vector control, transfection with *xCT*-Flag or *GPX4*-Flag partially but significantly reversed ZnSO_4_-induced suppression of viral gene expression (RT–qPCR; Fig. 7J), viral protein synthesis (Western blot; Fig. 7K) and infectious progeny yield (TCID_50_; Fig. 7L). This genetic rescue experiment provides direct evidence that zinc impairs ISKNV replication by targeting the xCT/GPX4 axis to induce ferroptosis.

### Zinc inhibits viral activity by inducing ferroptosis in vivo

**Fig. 8:**
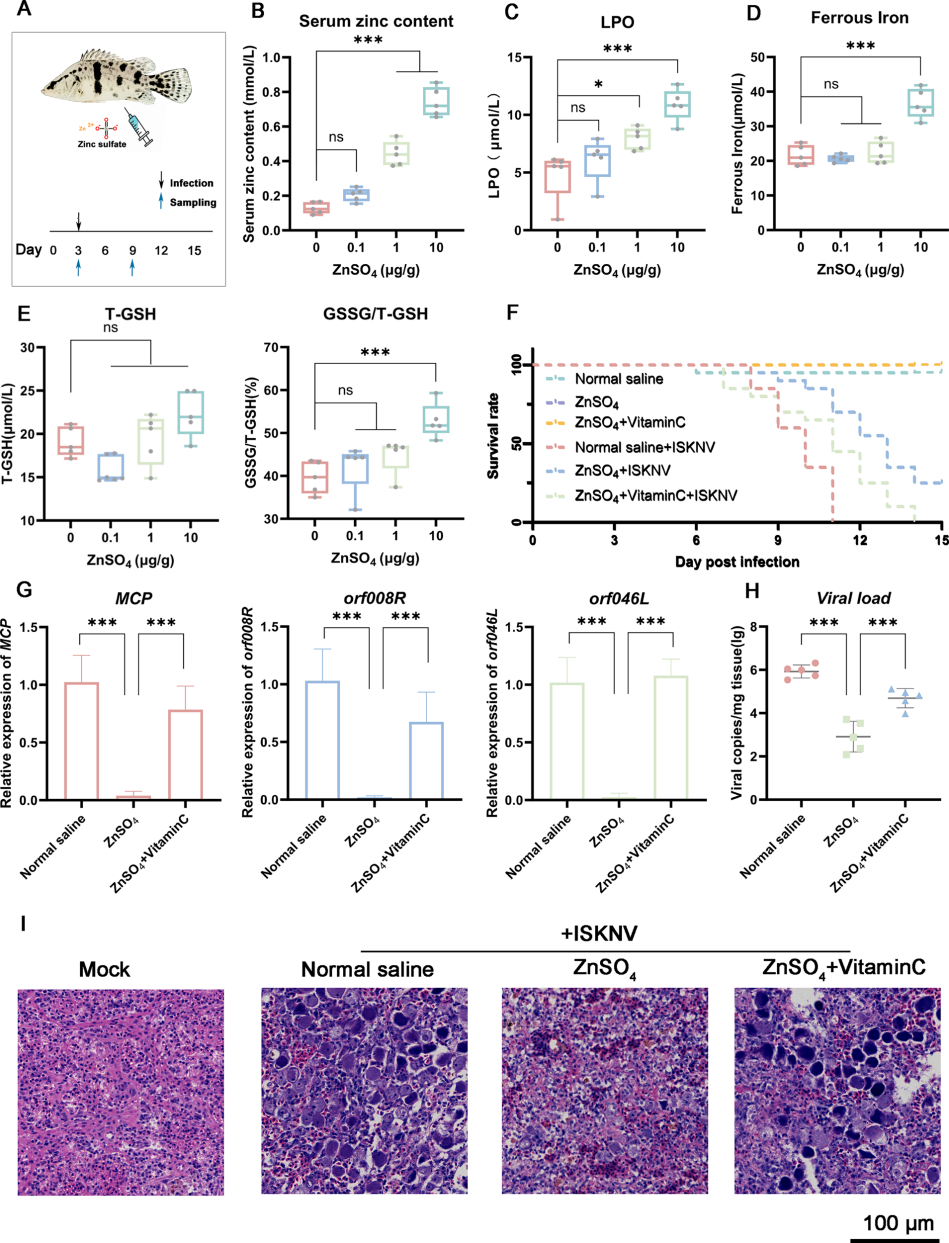
Zinc inhibits viral activity by inducing ferroptosis in vivo. **A** Schematic diagram of the experimental timeline and procedures. Zinc elevates systemic ferroptosis markers. Serum levels of zinc (**B**) LPO (**C**) Fe^2+^ (**D**) and glutathione (**E**) after ZnSO_4_ injection (*n* = 5). **F** Zinc improves survival in ISKNV-infected fish. Survival curves of fish pretreated with ZnSO_4_ ± vitamin C prior to ISKNV challenge. Normal saline served as solvent control. Uninfected fish served as treatment control (*n* = 20). Zinc reduces viral loads in the kidney. Viral gene expression (**G**) and titers (**H**) at 9 dpi (*n* = 5). Fish were pre-treated as in (**F**). **I** Zinc alleviates ISKNV-induced splenic injury. Hematoxylin and eosin (H&E) staining of spleen tissues from treated and infected fish at 9 dpi. Uninfected fish served as a blank control. Scale bar: 100 μm. Data are shown as mean ± SD. *P < 0.05; ***P < 0.001; *ns*, not significant.

To validate the physiological relevance of zinc-induced ferroptosis in antiviral defense, we administered graded doses of ZnSO_4_ (0.1, 0.5, 1.0 μg/g body weight) or saline control via intraperitoneal injection to *S. chuatsi* daily for three days (Fig. 8A). Intraperitoneal injection of ZnSO_4_ induced a dose-dependent increase in serum zinc levels (Fig. 8B), confirming systemic zinc bioavailability. Notably, at the 1 μg/g dose, key ferroptosis markers were significantly elevated: LPO, Fe^2+^, and GSSG surged versus saline controls, while total glutathione (T-GSH) remained unchanged (Fig. 8C–E). This biochemical profile establishes zinc’s capacity to induce ferroptosis in vivo.

Based on this threshold, all subsequent experiments employed 1 μg/g ZnSO_4_ with co-treatment of the ferroptosis inhibitor vitamin C (VC) (validated in *S. chuatsi*) [26]. After 3 days of pretreatment, fish were challenged with ISKNV and monitored for survival. The drug was well-tolerated in uninfected models, confirming its safety in the absence of viral infection. ZnSO_4_ monotherapy reduced cumulative mortality from 100% (saline control) to 75% and prolonged median survival time from 10 to 13 days. In contrast, VC and ZnSO_4_ co-treatment abrogated zinc’s protective effect, restoring mortality to 100% and reducing median survival time to 11 days (Fig. 8F). Virological and histopathological corroboration further solidified this mechanism. Consistent with survival outcomes, viral loads in the kidney were markedly reduced in ZnSO_4_-treated fish (vs. saline or ZnSO_4_ + vitamin C groups) (Fig. 8G, H). Histopathological analysis of spleens at 9 dpi provided direct tissue-level evidence: ZnSO_4_-treated fish exhibited significantly fewer enlarged cells (characteristic of ISKNV-induced cytopathology [21]), whereas saline and ZnSO_4_ + vitamin C groups showed extensive cellular enlargement (Fig. 8I). Notably, no pathological changes were observed in uninfected fish across all groups (Fig. S3), confirming that the observed cellular enlargement was specifically attributable to ISKNV infection. Collectively, these in vivo findings establish that zinc harnesses ferroptosis as a potent host defense mechanism to restrict ISKNV replication, reduce organ-specific viral burden, and ultimately delay mortality.

## Discussion

In this study, a novel antiviral mechanism was uncovered, wherein zinc ions exploit ferroptosis—a form of regulated cell death driven by iron-dependent lipid peroxidation—to combat ISKNV infection. We demonstrated that the influx of intracellular zinc ions triggered ferroptotic signaling through GPX4 suppression and lipid peroxide accumulation, independently of the Zn^2+^-MTF1 pathway. Moreover, ferroptosis induction functions as an innate host defense, as evidenced by both the promotion of ferroptosis by ISKNV infection and the direct antiviral effects of ferroptosis inducers. In vivo, the activation of Zn2+-mediated ferroptosis conferred significant survival benefits to infected hosts, reducing mortality by 25% and delaying disease progression, which was reversed by ferroptosis inhibitors. These findings renewed the understanding of zinc’s antiviral actions beyond classical paradigms and established ferroptosis as a therapeutically targetable pathway against aquatic viral diseases.

Zinc ions exhibit broad-spectrum antiviral activity through multiple established mechanisms [27], including direct virion destabilization in herpesviruses [6, 28] and inhibition of viral polyprotein processing in picornaviruses [6, 7]. While MTF1 serves as a principal cellular zinc sensor coordinating homeostatic responses like metallothionein expression [29], its involvement in zinc-mediated antiviral immunity remains undefined. Our study has conclusively demonstrated that while the MTF1 signal plays a crucial role in zinc homeostasis, it was not essential for zinc-mediated resistance to ISKNV activity. Instead, a novel defense axis was identified, in which zinc influx triggered ferroptosis and restricted viral replication. This mechanism produced phenotypic outcomes that aligned with the effects of zinc in defending against multiple pathogens, and revealed a previously unknown pathway that employed metal ion-mediated regulated cell death as a strategy for viral control.

Our findings that zinc inactivated GPX4 to execute ferroptosis were consistent with emerging evidence positioning zinc as a ferroptosis modulator. Traditional research has attributed the mechanism of zinc ion-mediated ferroptosis to the regulation of SLC39A14 and SLC39A7 [17, 30], which primarily control intracellular zinc flux [31]. Our results point to a more direct effector role of zinc ions. We observed that zinc treatment significantly suppressed both GPX4 expression and enzymatic activity, suggesting a direct interaction. This possibility was further supported by preliminary molecular docking analysis (Fig. S4), which indicated that Zn^2+^ could stably bind near the selenocysteine catalytic site of *sc*GPX4. This computational model provides a structural basis for our hypothesis, suggesting that zinc binding may sterically hinder substrate access or disrupt the catalytic triad essential for glutathione peroxidation. Mechanistically, zinc ions exhibit a high affinity for thiol/selenol groups, and the catalytic center of GPX4 depends on a conserved selenocysteine residue whose selenol group is critical for reducing lipid hydroperoxides [32, 33].

Therefore, we propose a testable model: zinc executes ferroptosis by directly binding to and inhibiting GPX4. While this computational model requires validation through site-directed mutagenesis and biophysical assays, it offers a novel mechanistic hypothesis. The xCT/GPX4 axis identified here is a core and evolutionarily conserved component of the ferroptosis machinery. Whether zinc exerts its antiviral effects via this same direct molecular axis in mammals constitutes an open and important question for future research. However, this extrapolation requires direct experimental validation in mammalian models, as species-specific differences in zinc metabolism and ferroptosis regulation cannot be ruled out. A pertinent example of such physiological divergence lies in the distinct biological context of teleost erythrocytes. Unlike the anucleated mammalian counterparts, fish erythrocytes are nucleated and retain functional organelles, including mitochondria [34]. This retention of cellular machinery endows them with the capacity for active gene transcription and the metabolic competence necessary to execute regulated cell death pathways, such as ferroptosis. This fundamental distinction elucidates our in vivo scRNA-seq findings, where stressed erythrocytes were identified as the major population undergoing ferroptosis-like changes, thereby highlighting their active and previously underappreciated role in the antiviral immune response.

The interplay between viral infections and ferroptosis exhibited context-dependent duality, as evidenced by our findings in ISKNV contrasting with established paradigms. Prevailing models demonstrated that viruses actively harness ferroptosis to enhance replication [35, 36]. For example, enterovirus upregulates ACSL4 to amplify lipid peroxidation for optimal virion assembly [37], while H1N1 swine influenza virus infection disrupts iron uptake and storage to enhance viral replication by inhibiting the system Xc^−^/GPX4 axis [38]. Conversely, we established that ISKNV-triggered ferroptosis functions counterintuitively as a host defense mechanism, imposing a replication barrier rather than facilitating dissemination. This functional divergence likely originates from fundamental differences in viral replication strategies. Lytic viruses might capitalize on ferroptosis-induced membrane fragility to promote progeny release, whereas iridoviruses including ISKNV [21], which depend on stable intracellular replication factories, suffer detrimental consequences from premature host cell death via ferroptosis. Critically, evidence was provided that ISKNV infection initiates ferroptosis. This represented a defensive repurposing of a viral-induced vulnerability into a host protective weapon, thereby recontextualizing ferroptosis as a context-dependent immune modulator.

This study demonstrated that zinc-based agents can effectively reduce the mortality rate and inhibit disease progression associated with ISKNV infection, offering a cost-effective strategy for disease prevention and control in aquaculture. Zinc additives are not only inexpensive but also widely utilized as feed supplements [39], which is particularly significant in the aquaculture sector. Moreover, the broad-spectrum antiviral properties of zinc highlight its potential for managing multiple pathogens in aquaculture systems. Additionally, the routine vitamin C supplementation widely used to optimize growth, health and stress resistance in aquatic animals [40] might need to be re-evaluated. The ferroptosis-inhibiting activity from vitamin C abolished zinc’s protection in challenged fish, implying excessive use of vitamin C may inadvertently facilitate the replication or activity of the virus.

## Materials and methods

### Animals, cell lines, and viruses

Mandarin fish (*Siniperca chuatsi*), less than one year old and 50 ± 10 g in weight, were purchased from a commercial fish farm in Foshan, Guangdong, China and were maintained in freshwater with continuous ventilation at 27 °C during the experiment. Given that there is no evidence of sex-dependent susceptibility to ISKNV in mandarin fish, fish were randomly assigned to groups regardless of sex. All fish were maintained in the laboratory for more than one week and were confirmed free of ISKNV by PCR prior to experiments.

Mandarin fish fry-1 (MFF-1) cell line was established by Dong et al. [41] and maintained in our laboratory. MFF-1 cells were cultured in Dulbecco’s Modified Eagle Medium (Cat. No. 12800082, DMEM; Gibco, NY, USA) supplemented with 10% fetal bovine serum (Cat. No. A5256701, FBS; Gibco) and maintained in a humidified atmosphere at 27 °C with 5% CO_2_. PCR analyses were performed to verify the species identity (mitochondrial DNA) and to ensure the absence of mycoplasma contamination.

The ISKNV strain (Genbank: OP896201.1) for in vitro and in vivo experiments was isolated from diseased mandarin fish in Foshan, Guangdong, China, in 2005 [42].

### Cell activity detection

MFF-1 cells were seeded in 96-well plates overnight and replaced with fresh DMEM with 10% FBS and different concentrations of zinc sulfate, zinc chloride, and zinc acetate for 72 h. Cell Counting Kit-8 (Cat. No. CK04, Dojindo, Mashiki-machi, Japan) was used to detect cell activity according to the manufacturer’s instructions.

### In vitro infection assay

MFF-1 cells were seeded in plates overnight before viral inoculation. For viral infection, cells were infected with ISKNV at a multiplicity of infection (MOI) of 1. These cells were incubated at 27 °C for 4 h to allow viral infection. Then the infectious medium was removed and replaced with fresh medium or medium containing certain compounds.

### RNA extraction and quantitative PCR for reverse transcription (RT–qPCR)

Cells were seeded in 24-well plates. At the sampling time point, the treated cells were lysed, and total RNA was extracted using Eastep® Super Total RNA Extraction Kit (Cat. No. LS1040, Promega, WI, USA). First-strand cDNA was synthesized from 200 ng of total RNA using TransScript® Uni All-in-One First-Strand cDNA Synthesis SuperMix for qPCR (Cat. No. AU341, TransGen Biotech, Beijing, China). RT–qPCR assays were conducted using RT–qPCR assays were conducted using SYBR Green *Pro* Taq HS (Cat. No. AG11701, Accurate Biology, Changsha, China), employing gene-specific primers as described in Table S1. To normalize the mRNA levels, *S. chuatsi β-actin* was used as the reference gene.

### Western blotting

Cells were cultured in 6-well plates. Following treatments and infection, the infected cells were lysed in 5X SDS-PAGE Sample Loading Buffer (Reducing) (Cat. No. AIWB-0025, Affinibody LifeScience, Wuhan, China), which was purchased from Guangzhou Boyu Biotechnology Co., Ltd. The lysates were heated in boiling water for 10 min and centrifuged at 12 000 g for 5 min. The supernatant was subjected to 4–20% FastPAGE^TM^ Precast Protein Gel (Cat. No. TSP024-12, Tsingke Biotech, Beijing, China) under 180 V for 45 min and transferred to Fluorotrans® W PVDF membranes (Cat. No. BSP0161, Cytiva, MA, USA). After being blocked in Ultra-low Background Sensitivity-enhancing Blocking Buffer (Cat. No. AFM-B500, Affinibody LifeScience), the membrane was probed with a mouse anti-isknvORF101L monoclonal antibody (mAb2D8, from Prof. Chuanfu Dong and Dr. Wenfeng Zhang) or GPX4 rabbit polyclonal antibody (Cat. No. ER1803-15, HUABIO, Hangzhou, China). To normalize the protein sample loading, α-Tublin or glyceraldehyde 3-phosphate dehydrogenase (GAPDH) from *S. chuatsi* served as a reference protein and was detected using mouse anti-α-Tubulin monoclonal antibody (Cat. No. 10005-M01, MaStar, Wuhan, China) or rabbit anti-GAPDH antibody (Cat. No. AB0037, Abways, Shanghai, China). Subsequently, the goat anti-mouse IgG (H + L) (Cat. No. W4021, Promega) or goat anti-rabbit IgG (H + L) (Cat. No. W4011, Promega) was employed as the secondary antibody incubated with the membrane. The signal was acquired from an Amersham^TM^ ImageQuant^TM^ 800 Western blot imaging system (Cytiva) with Chemistar^TM^ High-sig ECL Western Blotting Substrate (Cat. No. 180-5001, Tanon, Shanghai, China). Original, uncropped Western blot images for all data are included in the Supplementary Materials.

### TCID_50_ assay

Cells were cultured in 48-well plates. Following treatments and infection, these cells underwent repeated freezing and thawing to prepare samples awaiting detection. MFF-1 cells were seeded into 96-well plates and incubated overnight. Samples were serially diluted in culture medium, and each dilution was plated into eight wells. Plates were maintained at 27 °C for one week. Following incubation, cells were scored for presence or absence of cytopathic effects, and the Reed–Muench method was used to determine TCID_50_/mL.

### Dual-luciferase reporter assay

Cells were cultured in 24-well plates and replaced with fresh medium before transfection. The metal response element (MRE) luciferase reporter plasmid (pGL4.40-luc2P/MRE/Hygro, 0.4 μg) and pRL-TK (0.04 μg) plasmid were co-transfected into MFF-1 cells with Superluminal^TM^ High-efficiency Transfection Reagent (Cat. No. 11231804-10, MIKX, Shenzhen, China). After 36 h of transfection, cells were lysed in Passive Lysis 5X Buffer (Cat. No. E1941, Promega). Cell lysates were processed and assayed following the instructions of the Dual-Luciferase® Reporter Assay System (Cat. No. E1960, Promega).

### Measurement of cellular free zinc and serum zinc

MFF-1 cells were seeded in confocal dishes or 96-well plates with clear bottom black frames and treated for 24 h with zinc sulfate or TPEN (Cat. No. HY-100202, MCE, NJ, USA), a metal chelator with high affinity for zinc ions. Cellular free zinc was visualized by fluorescent probe Zinquin ethyl ester (Cat. No. 21253, AAT Bioquest, CA, USA). Cells were probed with 30 μM Zinquin fluorescent probes at 27 °C for 30 min in the dark. After washing thrice with PBS, cells were fixed in 4% paraformaldehyde for 30 min at room temperature. The nuclei were stained with propidium iodide (PI). Fluorescence signals were observed by a confocal microscope or an automatic microplate reader. The blood zinc levels were measured by a Blood Zinc Content Assay Kit (Cat. No. BC2815, Solarbio, Beijing, China) according to the manufacturer’s instructions.

### RNA-Seq

Cells were seeded into 6-well plates, cultured overnight to exceed 80% confluence, and then the culture medium was replaced with a medium containing 200 μM zinc sulfate for 72 h. The fresh medium was used as the blank control. Total RNA samples were collected using Trizol lysis. Following total RNA extraction, libraries were constructed according to the manufacturer’s instructions and sequenced on the Illumina NovaSeq X Plus platform.

### Transcriptome data processing

fastp v0.24.0 [43] was using with the parameters “--detect_adapter_for_pe --dont_eval_duplication --cut_front --cut_tail --cut_right --cut_front_window_size 1 --cut_front_mean_quality 5 --cut_tail_window_size 1 --cut_tail_mean_quality 5 --cut_right_window_size 5 --cut_right_mean_quality 20 --length_required 50 --qualified_quality_phred 20 --unqualified_percent_limit 30” to preprocess and perform quality control on raw data. The resulting clean data were aligned to the mandarin fish reference genome (GenBank: GCA_020085105.1, RefSeq: GCF_020085105.1) using HISAT2 v2.2.1 [44]. Then, gene expression levels were quantified using SAMtools v1.22.1 [45] and StringTie v3.0.0 [46]. Principal component analysis (PCA) and intra-group correlation analysis were performed on the samples in R v4.5.1. For differential expression gene (DEG) identification, DESeq2 v1.49.2 [47] was used with the criteria of |log2(FoldChange)| > 1 and padj < 0.05 to identify DEGs. Subsequently, clusterProfiler v4.17.0 [48] was used for enrichment analysis of DEGs, with KEGG pathway enrichment analysis to identify differences in related molecules or metabolic pathways, and gene set enrichment analysis (GSEA) to evaluate whether predefined KEGG pathway gene sets exhibited statistically significant and consistent expression differences between the control and infection groups. Statistical significance in all enrichment analyses was defined as *P* < 0.05.

### Examination of enzyme activity

The levels of MDA, GPX4, LPO, GSH/GSSG, and ferrous iron were determined via the following kits from Elabscience (Wuhan, China): Enhanced Cell Malondialdehyde (MDA) Colorimetric Assay Kit (Cat. No. E-BC-K814-M), Glutathione Peroxidase 4 (GPX4) Activity Assay Kit (Cat. No. E-BC-K883-M), Lipid Peroxide (LPO) Fluorometric Assay Kit (Cat. No. E-BC-F003), Total Glutathione (T-GSH)/Oxidized Glutathione (GSSG) Colorimetric Assay Kit (Cat. No. E-BC-K097-M), and Ferrous Iron Colorimetric Assay Kit (Cat. No. E-BC-K773-M).

### Transmission electron microscopy

Cells were seeded in 60 mm diameter dishes, cultured overnight to exceed 80% confluence, and then the culture medium was replaced with a medium containing 200 μM zinc sulfate for 72 h. Fresh medium and dimethylsulfoxide (DMSO) served as the blank control and solvent control, respectively. The cells were washed with PBS, followed by trypsin digestion and centrifugation to pellet the cells. The cells were resuspended in 2.5% glutaraldehyde and fixed at room temperature for 30 min in the dark, and the fixed cells were subsequently transferred to 4 °C for storage. The fixed cell samples were commissioned by Wuhan Sevier Biotechnology Co., LTD for subsequent stepwise dehydration, embedding, polymerization and staining.

### Single-cell transcriptome sample collection, library preparation, and sequencing

Before the experiment, mandarin fish were randomly divided into two groups: the infection group was intraperitoneally injected with 100 µL of ISKNV at 5 × 10^5^ TCID_50_/mL, while the control group was injected with an equal volume of sterile PBS. Seven days post-infection, fish were euthanized using MS-222, and their spleens were collected, washed twice with sterile PBS, and stored in tissue preservation solution. To minimize inter-individual variation, spleens from five fish per group were pooled into a single sample after tissue dissociation, and libraries were constructed following the manufacturer’s (10× Genomics) protocol, followed by sequencing on the Illumina NovaSeq X Plus platform.

### Single-cell transcriptome data processing

Raw sequencing data were aligned to the mandarin fish reference genome using Cell Ranger v9.0.1 to generate expression matrices, which were then processed in Seurat v5.3.0 [49] for further analysis. Only cells with mitochondrial gene content < 10%, unique molecular identifiers (UMIs) < 15,000, and detected genes between 350 and 3500 were retained. DoubletFinder v2.0.6 [50] was used to remove doublet contamination from both samples, followed by merging the expression matrices of all samples in Seurat and normalizing them using the “LogNormalization” method. The FindVariableFeatures function was used to identify the top 2000 highly variable genes. Expression data for these genes were standardized, scaled, and centered using the ScaleData function. Principal component analysis (PCA) was performed on these genes, and the top 30 principal components were integrated across samples using the Harmony function to correct for batch effects. The top 30 Harmony dimensions were used for clustering and tSNE analysis with the FindNeighbors and RunUMAP functions. Cell clusters were identified using the FindClusters function with a resolution of 0.5. Differentially expressed genes (DEGs) between clusters were determined using the FindAllMarkers function with the Wilcoxon rank-sum test. Cell cluster identities were assigned based on canonical marker genes, and clusters assigned to the same cell type were merged. For DEGs, the filtering criteria were set as avg_log2FC > 0.3, p_val_adj < 0.05, and expression in at least 15% of cells. KEGG pathway enrichment analysis was performed on DEGs using clusterProfiler v4.17.0. Biological process scores were calculated using the AddModuleScore function in Seurat. Pseudotime analysis was conducted using Monocle v2.37.0 [51] based on the union of the 2000 most variable genes in an unsupervised manner, with dimensionality reduction performed using the discriminative dimensionality reduction with trees (DDRTree) algorithm.

### Animal challenge assay

*S. chuatsi* individuals were given daily intraperitoneally with different kinds of activators, inhibitors or normal saline for three days prior to challenge. For viral propagation, fish were injected with 100 µL of 1 × 10^6^ copies/mL of ISKNV. Animal mortality in each group was recorded each day and finally calculated. For viral load analysis, the metanephric kidney or spleen was harvested and ground on the indicated days, and viral load was calculated by RT–qPCR and absolute quantitative PCR.

### Histopathological analysis

Hematoxylin and eosin (H&E) staining of spleen tissues from treated and infected fish at 9 dpi. Uninfected fish served as a blank control. The samples were fixed with 4% paraformaldehyde for 24 h at room temperature. The fixed samples were commissioned by Wuhan Sevier Biotechnology Co., LTD for sectioning and H&E staining. The slides were observed under a microscope.

### Prediction and analysis of protein–metal interactions

The interaction structure between *sc*GPX4 and zinc ions was predicted using the AlphaFold Server [52]. The template date was set to February 3, 2025, with the random seed set to “Auto”. Interactions between the protein and zinc ions within the predicted structure were calculated using Arpeggio [53]. Structural visualization was performed using PyMOL v3.1.6.1.

### Statistical analysis

No statistical methods were used to predetermine sample size. Sample sizes were chosen based on previous experience and similar studies to ensure adequate statistical power. No data were excluded during the analysis process. The investigators were not blinded to the group allocation during the experiment and outcome assessment. However, all samples and animals were processed using standardized procedures to minimize bias. Statistical analysis was performed with GraphPad Prism v8.0 software. Data are presented as mean ± standard deviation (SD). The normality of data distribution was assessed using the Shapiro-Wilk test, and homogeneity of variance was verified using the Brown-Forsythe test. For comparisons between two groups, a two-tailed Student’s *t*-test was used. For multiple group comparisons, one-way analysis of variance (ANOVA) followed by Tukey’s post hoc test was performed. Survival analysis was conducted using the Log-rank test. The median lethal time (LT_50_) was calculated by the Probit analysis using SPSS Statistics.

## Supplementary information

Supporting Information

Original Data Western Blots

## Data Availability

The RNA-seq and scRNA-seq data generated in this study have been deposited in the NCBI GEO database under accession numbers GSE318614 and GSE318615. All other data supporting the findings of this study are available within the article and its supplementary materials.
