# Supplementary material for: Zinc ions attenuates iridovirus infection through regulation of ferroptosis pathways
Source: Cell Death Discov. 2026 Apr 20;12:260. doi: 10.1038/s41420-026-03114-x (PMC13223261; doi:10.1038/s41420-026-03114-x)
Supplement: Supplementary file 1 — Supporting Information [file 41420_2026_3114_MOESM1_ESM.docx]

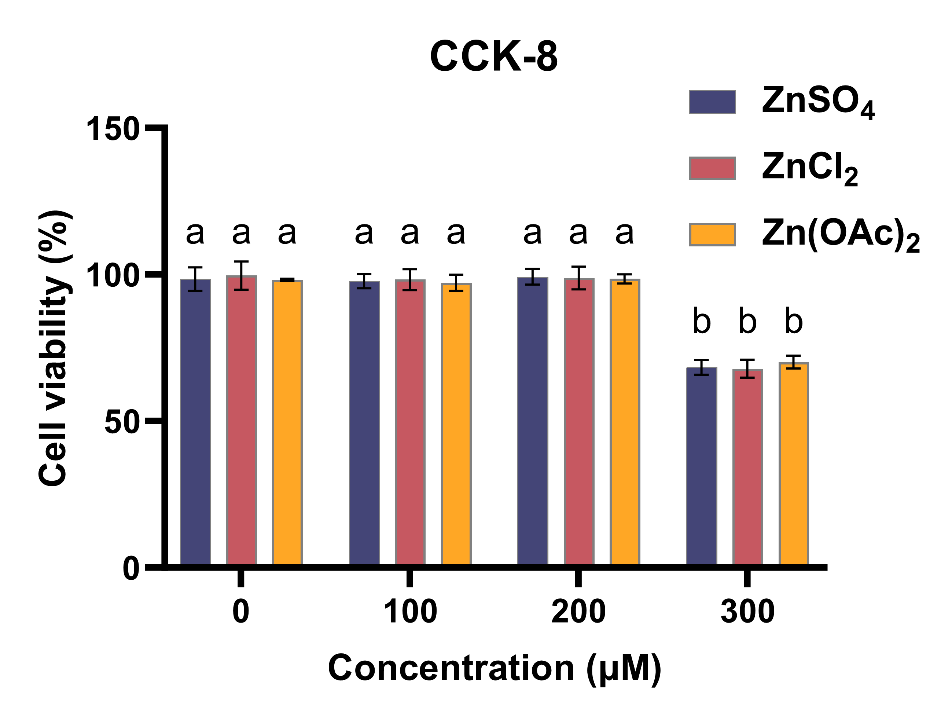

**Figure S1** Effects of different concentrations of zinc on the cell viability of MFF-1 cells after 72 h of culture (*n* = 5). Different lowercase letters indicate a significant difference (*P* < 0.01).


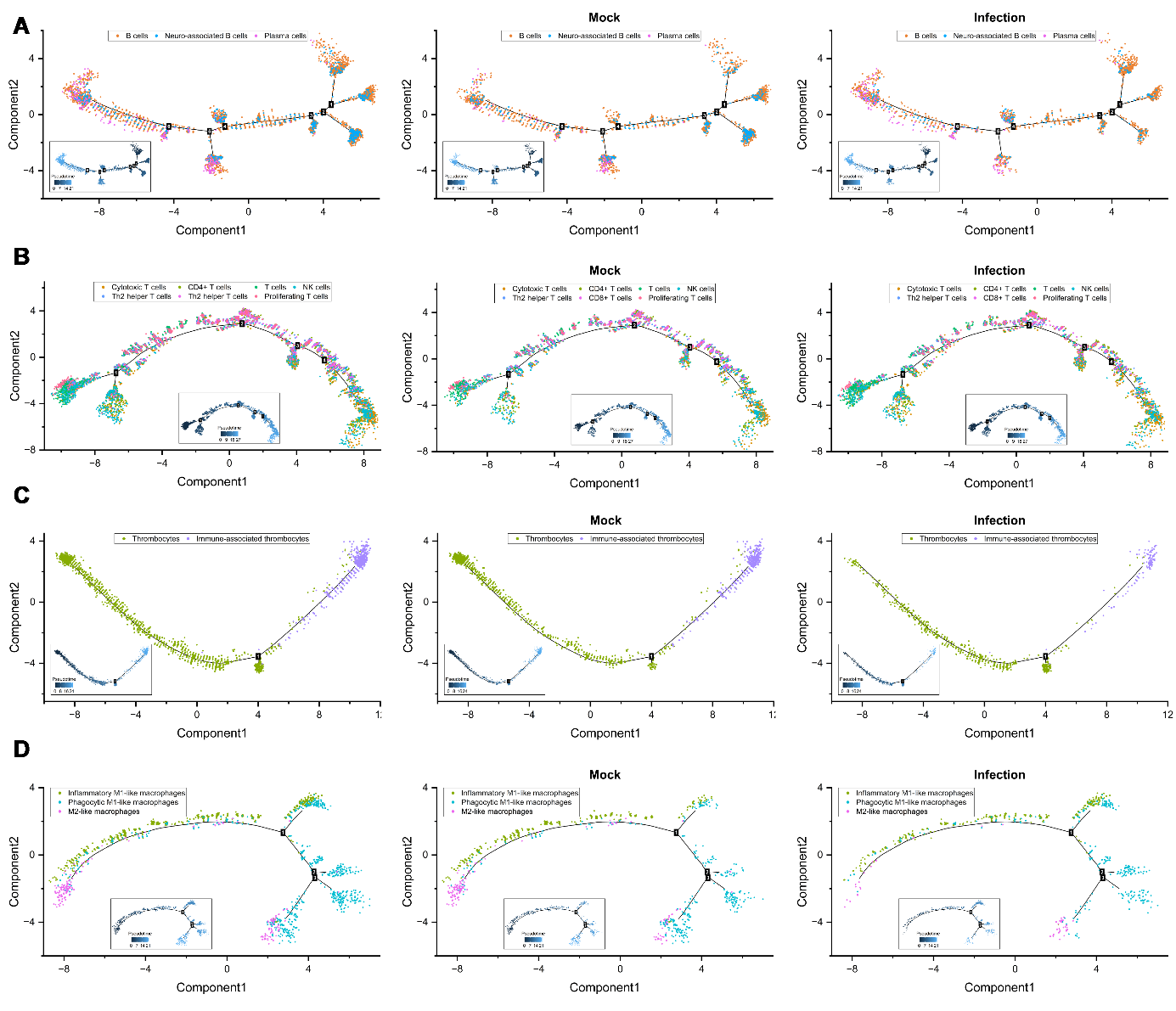


**Figure S2.** Pseudotime analysis of cells in the spleen of mandarin fish after ISKNV infection. (A–D) Pseudotime analysis of B cells (A), T cells (B), thrombocytes (C), and macrophages (D) in the spleen of control and infected mandarin fish, each dot represents a cell, and each color represents a cell cluster.


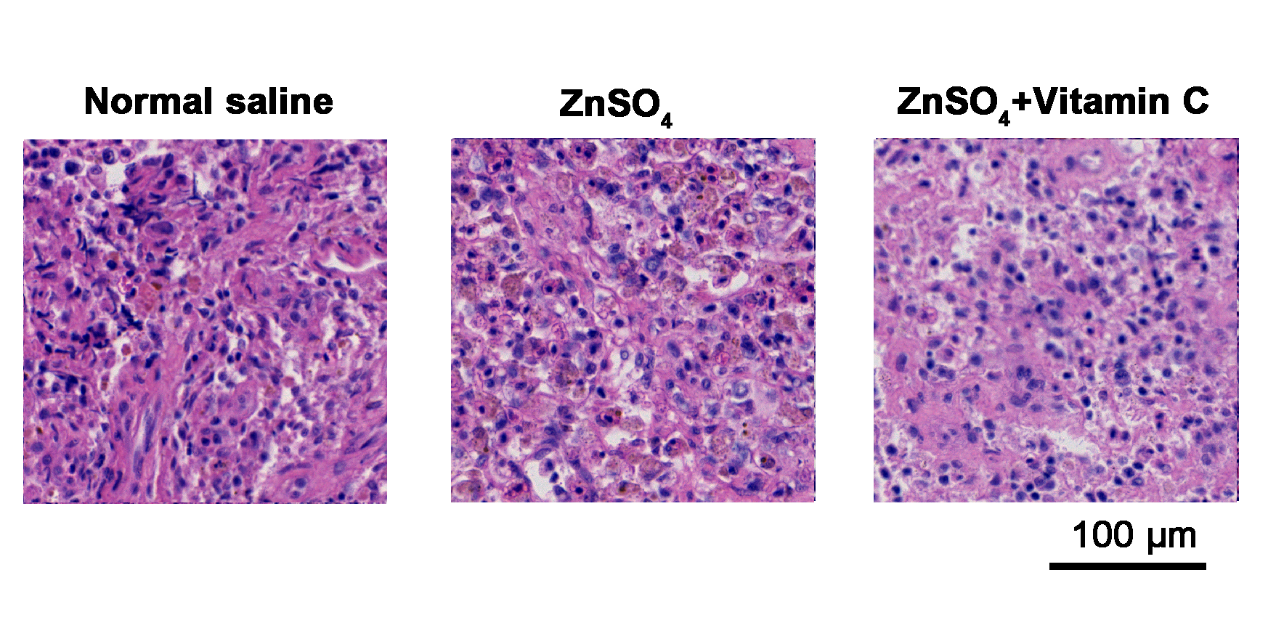
**Figure S3** Histological examination of spleen tissue by hematoxylin and eosin (H&E) staining. Scale bar = 100 μm.


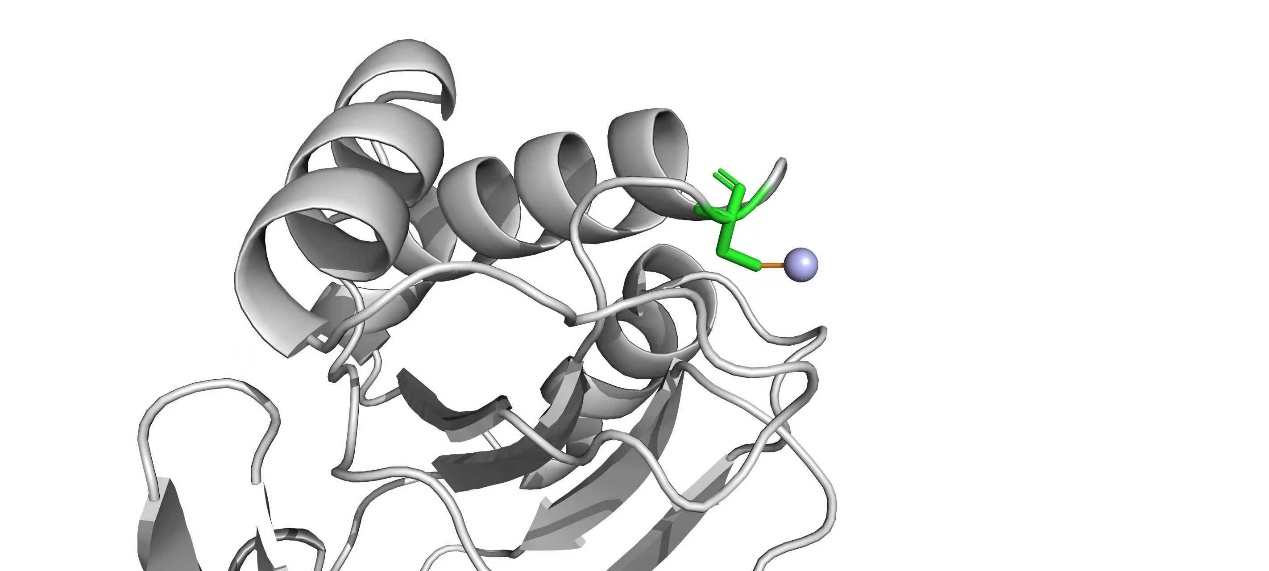


**Figure S4** Predicted interaction structure between *sc*GPX4 and zinc ions. Metal complex interactions (orange) are observed between residue 76 of *sc*GPX4 (green) and the zinc ion (light blue).

**Table S1.** Primers used in this study.

| Name | Experiment | Sequences (from 5’ to 3’) |
| --- | --- | --- |
| ISKNV-F | PCR detection | ATGTCTGCAATCTCAGGTGCAAACG |
| ISKNV-R | PCR detection | TTACAGAGGGAAGCCTGCGGCGCCG |
| MRV-F | PCR detection | ATGTCTTCTGTTACGGGTTCTGGC |
| MRV-R | PCR detection | TTACAGGATGGGGAAACCCATG |
| SCRV-F | PCR detection | ATGGAAAACCAAATCATCAAGAG |
| SCRV-R | PCR detection | TCACAAAGCTTGGTGTTTCAG |
| isknvMCP-F | qPCR and RT-qPCR | CAATGTAGCACCCGCACTGACC |
| isknvMCP-R | qPCR and RT-qPCR | ACCTCACGCTCCTCACTTGTC |
| isknvORF008R-F | RT-qPCR | TGACCTGTGGCCTAGATGATAAC |
| isknvORF008R-R | RT-qPCR | AGAGGCAGAGCAGCAGCATGTAGAGT |
| isknvORF046L-F | RT-qPCR | TGGCAAATGCGGTGCTATGA |
| isknvORF046L-R | RT-qPCR | TGGCCCCAGCGTTTGGTAT |
| *sc*MTF1-F | RT-qPCR | ATCACCATAGCACCCACCC |
| *sc*MTF1-R | RT-qPCR | GCCAGCATTTCCTCCCAC |
| *sc*MT2-F | RT-qPCR | ATGGACCCTTGCGACTG |
| *sc*MT2-R | RT-qPCR | ACACCAGCTGCTGTAAGTGA |
| *sc*ZnT1a-F | RT-qPCR | CCCAAGCAAATCAACATACA |
| *sc*ZnT1a-R | RT-qPCR | GATGCCCTCGTTGTGAAA |
| *sc*ZnT8-F | RT-qPCR | CCCAACCAGGAGGCATTA |
| *sc*ZnT8-R | RT-qPCR | CGTCTGTCATCACCGCAAG |
| *sc*GPX4-F | RT-qPCR | GGATGATCCAAGTGTGGTGGA |
| *sc*GPX4-R | RT-qPCR | ATCCACTGGTGCTTGGAGAG |
| *sc*SLC7A11-F | RT-qPCR | TAACCCTGAACGGACTGTGC |
| *sc*SLC7A11-R | RT-qPCR | GTAGTACGCCACGTTGGTCA |
| *sc*ASCL1a-F | RT-qPCR | ACATCGGTTCTGCTCTTCTTC |
| *sc*ASCL1a-R | RT-qPCR | CTTCACTGTTCGTCCTTTTCC |
| *sc*Ferritin-F | RT-qPCR | ATCAACAAACTCATCAACCTCAA |
| *sc*Ferritin-R | RT-qPCR | ACCACCTCTCCAATCCTCTCTAC |
| *sc*β-actin-F | RT-qPCR | CCCTCTGAACCCCAAAGCCA |
| *sc*β-actin-F | RT-qPCR | CAGCCTGGATGGCAACGTACA |
| siMTF1-1 | siRNA | GGACGACGAUGAAGAAGAATT |
| siMTF1-2 | siRNA | GGGCGACUAUGUACUUUGATT |
| non-targeting control siRNA | siRNA | GGUCAUCGAUGGACAUAAATT |
