# Supplementary figures and images for: Zinc ions attenuates iridovirus infection through regulation of ferroptosis pathways
Source: Cell Death Discov. 2026 Apr 20;12:260. doi: 10.1038/s41420-026-03114-x (PMC13223261; doi:10.1038/s41420-026-03114-x)

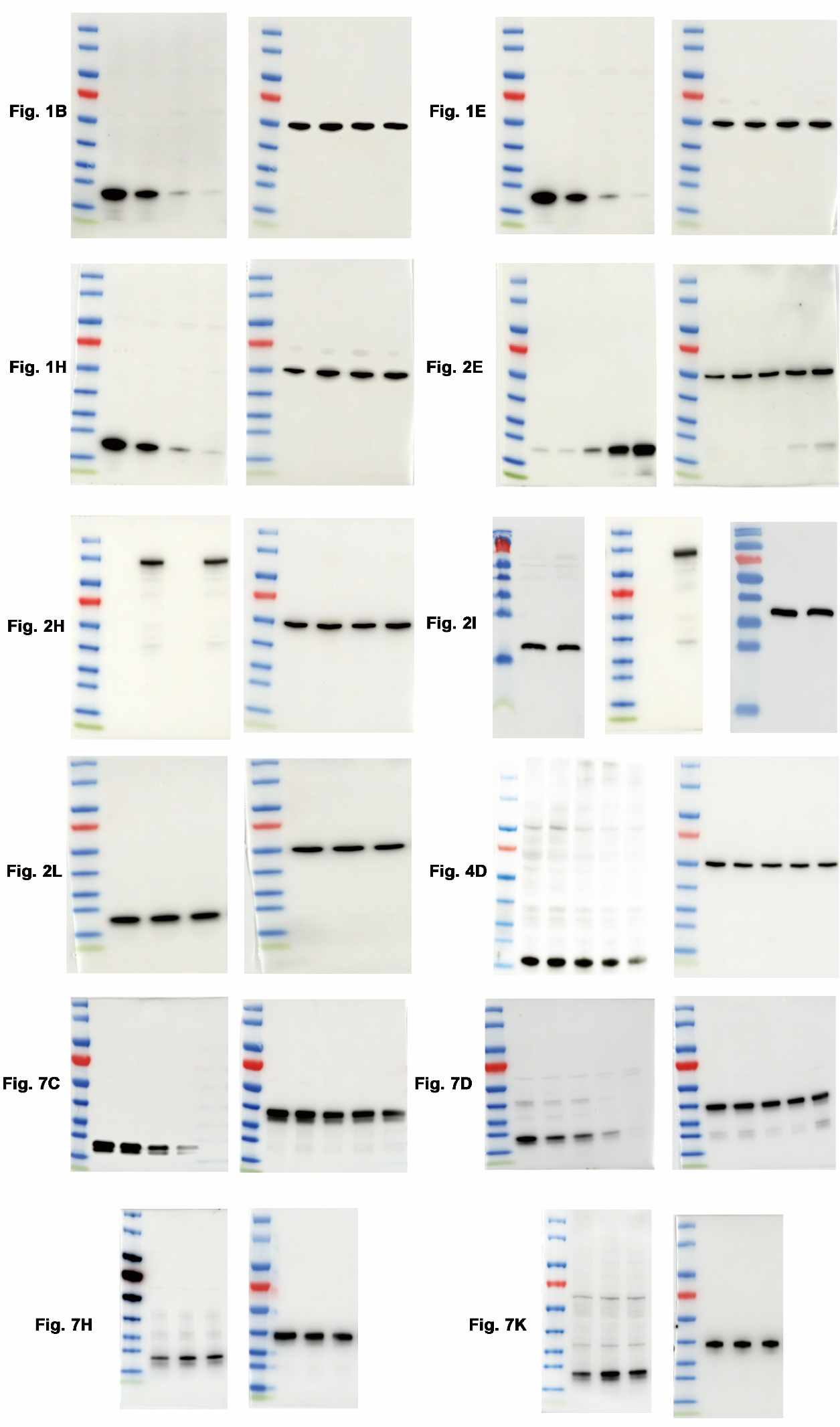

Supplement: Supplementary file 2 — Original Data Western Blots [file 41420_2026_3114_MOESM2_ESM.docx]
